# The relationship between impulsivity and non-suicidal self-injury in adolescents: the chain-mediated effects of parenting style and distress tolerance

**DOI:** 10.3389/fpsyt.2026.1784109

**Published:** 2026-04-16

**Authors:** Hong Chen, Yuyu Gu, Congwen Yang, Hui Xiang

**Affiliations:** 1 School of Psychology, Guizhou Normal University, Guiyang, Guizhou, China; 2 Department of Psychology, Guizhou Provincial People’s Hospital, Guiyang, Guizhou, China

**Keywords:** adolescents, distress tolerance, impulsivity, non-suicidal self-injury, parenting style

## Abstract

**Objective:**

The purpose of this study is to explore the related risk factors and protective factors of adolescent non suicidal self injury (NSSI).

**Methods:**

Utilizing the experience sampling method, we recruited 311 adolescents engaging in NSSI, all without other mental disorders, from five public high schools in a specific city. Questionnaire surveys were administered, employing the Chinese version of the Short-Form Egna Minnen av Barndoms Uppfostran (s-EMBU-C), Distress Tolerance Scale-Revised (DTS-CR), Adolescent Self-harm Behavior Questionnaire, Barratt Impulsiveness Scale version 11 (BIS-11).

**Results:**

1) The findings indicate that NSSI in adolescents is positively correlated with impulsivity and negative parenting styles (*P* < 0.01), while it is negatively correlated with distress tolerance and positive parenting styles (P<0.01). Impulsivity is negatively correlated with distress tolerance and positive parenting styles (*P* < 0.01) and positively correlated with negative parenting styles (*P* < 0.01). Furthermore, distress tolerance is negatively correlated with negative parenting styles (*P* < 0.01) and positively correlated with positive parenting styles (*P* < 0.01). 2) This study reveals that both negative and positive parenting styles serve as complete mediators in the relationship between impulsivity and NSSI behavior in adolescents, with distress tolerance as a significant factor.

**Conclusion:**

Impulsivity significantly influences NSSI behavior in adolescents through the mediation of parenting styles (both negative and positive) and distress tolerance.

## 1 Introduction

Non-suicidal self-injury (NSSI) is a prevalent behavior among adolescents. A meta-analysis conducted in 2017 revealed that the overall prevalence rate of NSSI among Chinese secondary school students was 22.37% (1). According to Erikson’s theory of psychosocial development, adolescence is generally defined as occurring between the ages of 12 and 18, with the primary developmental task being the establishment of “self-identity.” This process involves addressing questions such as “Who am I?” and “What is my position in society?” (2). In contrast, Piaget posited that adolescents enter the stage of formal operations. While they develop the capacity for abstract logical thinking, this ability can also result in “self-centeredness” and the phenomenon of an “imaginary audience.” These traits are, to some extent, associated with the adventurous behaviors and emotional distress commonly experienced by teenagers (3). NSSI represents a form of NSSI that poses risks of scarring and infection, and it is frequently linked to an increased risk of suicide (4). Thus, understanding the psychological mechanisms underlying these relationships is crucial for developing effective intervention strategies. In this study. Linehan’s bio-social model of NSSI asserts that the interplay between inherent predisposing factors and maladaptive environmental influences during development results in emotional dysregulation in individuals (5), Emotional dysregulation (ED) is characterized by the inability to manage and organize emotions effectively, leading to responses and difficulty returning to baseline levels (6). Distress tolerance denotes an individual’s capacity to withstand negative internal states and emotional distress (7, 8). Research indicates that emotional dysregulation is significantly negatively correlated with distress tolerance. The regulation of emotions serves as a primary function of NSSI (9). Prior studies have established a significant correlation between NSSI and impulsivity (10, 11). Investigations utilizing object interference tasks among individuals with NSSI indicated that those in the recurrent NSSI group demonstrated deficits in control during tasks related to the regulation of NSSI behavior (12). An “ineffective environment” denotes an adverse setting that develops later in life, primarily characterized by parental emotional abuse and neglect. Research indicates that an ineffective family environment during childhood is a significant factor contributing to the emergence of NSSI (5). Among these factors, parenting styles exhibit a notable correlation with the occurrence, recurrence, and severity of NSSI(C. 13).

Studies have shown that distressing emotions in children and adolescents are significantly negatively correlated with positive parenting styles (14). As a susceptibility factor, the risk associated with impulsivity requires activation and transformation through a negative family environment, while a positive family environment can mitigate this risk (15). However, the existing findings do not clarify the mechanisms or conditions under which impulsivity manifests as specific NSSI. The diathesis-stress model suggests that an individual’s inherent qualities do not directly cause the disorder; rather, they are activated by stressful life events, which in turn initiate adverse psychological processes that ultimately influence problematic behavior (16).

To elucidate the key transmission mechanism from traits to behaviors, we constructed and tested a chain model that begins with impulsivity. In this model, impulsivity is posited to first predict parental parenting styles, which subsequently predict the core mechanism of distress tolerance, ultimately correlating with the risk of NSSI. This model aims to empirically examine the specific pathways through which innate susceptibility factors, as outlined in biosocial models, exert influence via postnatal environmental and emotional regulation processes. Crucially, the diathesis-stress model implies that inherent vulnerabilities such as impulsivity should not exert a direct effect on behavioral outcomes like NSSI. Instead, their influence is hypothesized to be indirect, operating through their interaction with environmental stressors and subsequent psychological processes. Applied to the present study, this theoretical perspective provides the foundation for expecting that the relationship between impulsivity and NSSI is fully mediated by parenting styles and distress tolerance. In other words, impulsivity is theorized to have no direct association with NSSI after accounting for these mediators—a prediction that distinguishes the current model from previous research that has often assumed or tested only direct effects.

**Figure 1:**
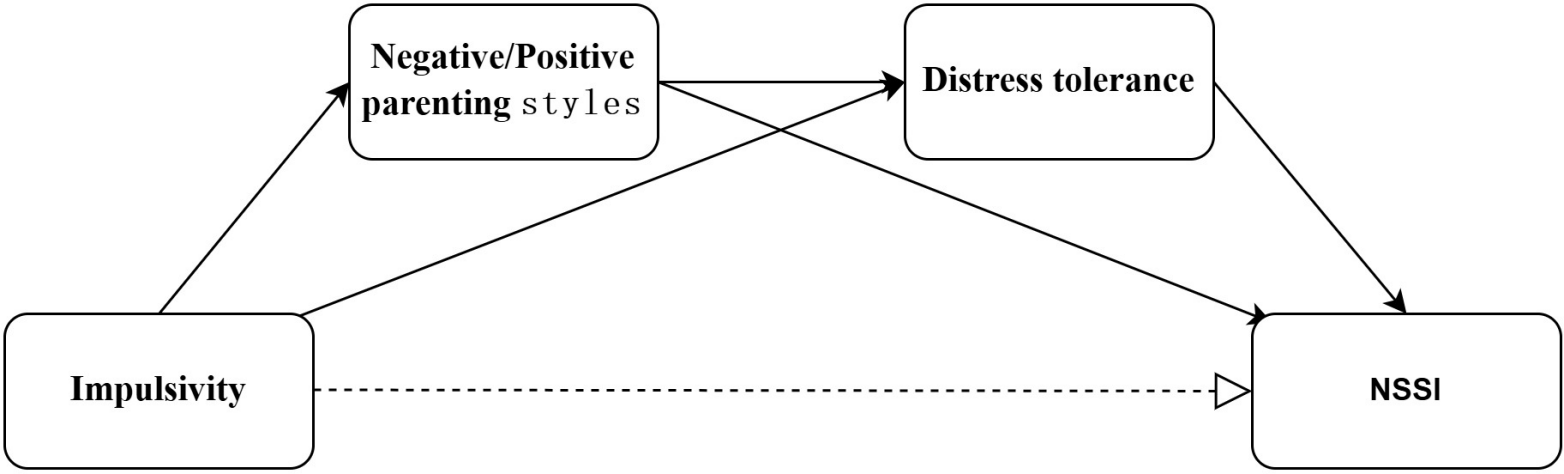
Conceptual framework diagram.

Grounded in this theoretical framework, we propose the following hypotheses: (1) Impulsivity is indirectly associated with NSSI through the sequential mediating effects of parenting styles and distress tolerance; (2) After accounting for these mediators, there is no direct association between impulsivity and NSSI, indicating full mediation (**Figure 1**). 

## 2 Methods

### 2.1 Participants

This study focused on adolescents engaging in NSSI without comorbid mental disorders. NSSI frequently coexists with other mental disorders (17), In prior studies, participants with NSSI were identified based on scores, and other comorbid mental disorders were not systematically excluded. To address this limitation and clarify the pathological and psychological mechanisms underlying NSSI, the present study systematically excluded individuals with comorbid mental disorders.

#### 2.1.1 Inclusion criteria

① Participants were aged between 12 and 18 years; ② Screening utilized the Adolescent Self-Harm Scale, with adolescents who reported self-harm behavior more than five times per year classified as positive cases. Following this, two psychiatrists at the attending physician level or above conducted structured clinical interviews to establish diagnoses in accordance with DSM-5 criteria for NSSI(Diagnostic criteria are provided in the DSM-5); ③ Informed consent and voluntary participation were secured; ④ Participants had never received pharmacological treatment.

#### 2.1.2 Exclusion criteria

Diagnoses according to DSM-5 included: ① Attention-Deficit/Hyperactivity Disorder (ADHD), with or without hyperactivity; ② Depressive disorders, anxiety disorders, and other major mental disorders; ③ Substance-related and addictive disorders (18).

Based on general multivariate statistical analysis principles and established statistical guidelines, the sample size is calculated as the number of study variables multiplied by a factor of 5 to 10. In this study, 18 variables are examined. Accounting for a 20% attrition rate, the minimum required sample size ranges from 108 to 216 cases.

### 2.2 Procedure

**Figure 2:**
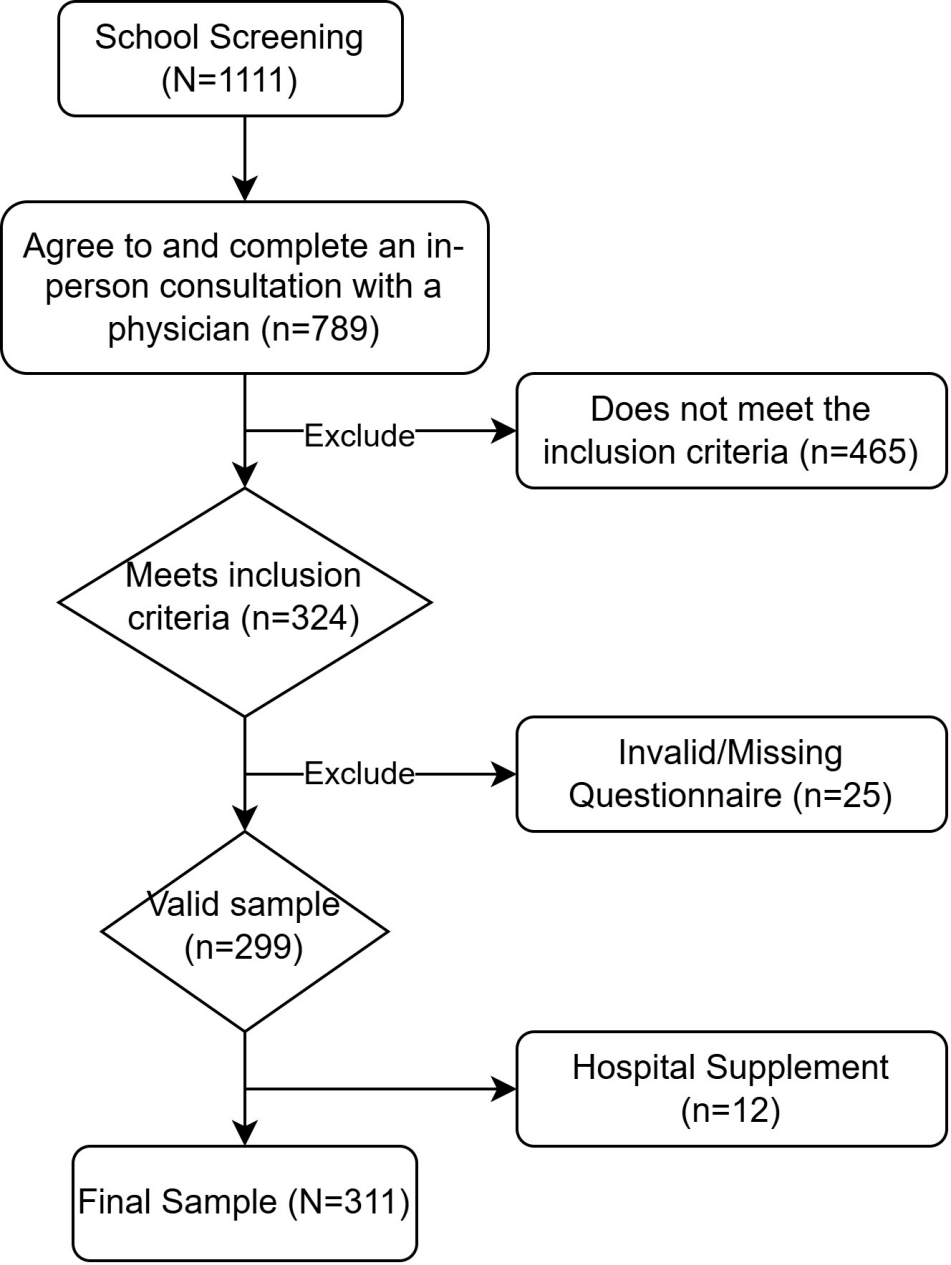
Sample recruitment flowchart.

From November 2022 to December 2023, a total of 1,111 adolescents at risk for self-harm were initially screened using the “Adolescent Self-Harm Behavior Questionnaire” across five middle schools in Guiyang. Of these, 789 students and their parents consented to and completed subsequent semi-structured interviews with clinical doctors. Based on the diagnostic criteria outlined in the DSM-5 and additional exclusion criteria, 324 adolescents were identified as individuals with NSSI who met the research conditions. Following the collection of their complete psychological assessment questionnaires, those with 25 or more invalid responses or a significant number of missing items were excluded. Additionally, 12 adolescents with NSSI who met the same criteria were recruited through hospital channels. Consequently, a total of 311 valid samples were ultimately included in the data analysis. The sample screening process is illustrated in **Figure 2**.

This research has received approval from the Medical Ethics Committee of Guizhou Provincial People’s Hospital (Lun Shen [Research] No. 2023-124).

### 2.3 Measures

#### 2.3.1 Sociodemographic information form

The General Information Questionnaire collected critical socio-demographic variables from participants, including gender, grade, only-child, whether the child comes from a divorced family, and the parents’ years of education. All participants were recruited from local public middle and high schools, encompassing students from Grades 7 to 12. This standardized instrument was developed to gather essential socio-demographic data using structured items.

#### 2.3.2 Barratt impulsiveness scale version 11

This study utilized the Barratt Impulsiveness Scale version 11, revised by Zhou Liang et al. (19). The scale consists of 26 items rated on a 4-point scale: rarely, occasionally, often, and always. It assesses three dimensions of impulsivity: attentional, motor, and non-planning. The overall Cronbach’s α for the scale was 0.76, while the dimension-specific Cronbach’s α coefficients were 0.56, 0.66, and 0.69, respectively.

#### 2.3.3 Adolescent self-harm behavior questionnaire

This study utilized the Adolescent Self-harm Behavior Questionnaire, revised by Feng Yu et al. (20), to evaluate self-harm behavior, The questionnaire consists of 19 items, including 18 closed-ended questions and one open-ended question. This instrument primarily examines the methods, frequency, and severity of self-harm behaviors. Self-harm frequency is assessed on a 0–3 scale, while self-harm severity is evaluated on a 0–4 scale. Each item’s score is derived by multiplying the frequency of self-harm by its corresponding severity. The total score for the questionnaire is obtained by summing the scores of all 19 items. The Cronbach’s α coefficient for the complete scale is 0.92.

#### 2.3.4 Distress tolerance scale-revised

The measurement of distress tolerance utilizes the Distress Tolerance Scale-Revised, developed by Liu Xiaolin et al. (21). The DTS-CR consists of 13 items categorized into three dimensions: tolerance, regulation, and appraisal. Respondents rate their agreement using a 5-point scale, where 1 indicates “Strongly disagree” and 5 indicates “Strongly agree.”

#### 2.3.5 The Chinese version of the short-form Egna Minnen av Barndoms Uppfostran

To assess parenting styles, s-EMBU-C which revised by Jiang et al. (22) was employed. This 42-item scale includes two subscales for fathers and mothers, each covering three dimensions: overprotection, rejection, and emotional warmth. Positive parenting styles are determined by the average score of emotional warmth from both parents, while negative parenting styles are assessed based on the average scores of rejection and overprotection from both parents. The scale employs a 4-point rating system, with 1 signifying “never” and 4 signifying “always.” The Cronbach’s α coefficients for both parents across the three dimensions ranged from 0.74 to 0.84.

### 2.4 Statistical analyses

Data were organized and analyzed utilizing SPSS 22.0. The chained mediating effects of parenting styles and distress tolerance were assessed using Model 6 of the SPSS PROCESS 2.16 macro program.

### 2.5 Common method deviation test

This study employed the marker variable technique to assess common method bias (CMB), following the procedures recommended by Lindell and Whitney (23) and Podsakoff et al. (24). Father’s educational level was selected as the marker variable based on both theoretical and empirical grounds. Theoretically, within Bronfenbrenner’s ecological systems framework, parental education is conceptualized as a distal environmental factor that influences adolescent development indirectly through proximal processes such as parenting practices, rather than exerting direct effects on psychological traits (25). This theoretical distinction is supported by empirical evidence: previous research has demonstrated that parental educational attainment has minimal direct effects on adolescent psychological characteristics, with associations largely mediated by family processes (26).

Analysis procedure: First, we examined the correlations between the marker variable and all substantive variables. Results showed that father’s education was not significantly correlated with impulsivity, negative parenting, distress tolerance, or NSSI (all p > 0.05), confirming its empirical distinctness from these core psychological constructs. The weak correlation with positive parenting (r = -0.217, p < 0.001) is theoretically plausible, as parental education may relate to parenting practices, and does not disqualify it as a marker variable (23). Second, to evaluate the influence of CMB on our findings, father’s educational level was included as a covariate predicting all endogenous variables in the two chain mediation models. We then compared the path coefficients and significance levels between the original models and the models controlling for father’s education.

After controlling for father’s educational level, all originally significant path coefficients remained significant, and the direction of all effects remained unchanged. The absolute changes in path coefficients ranged from 0 to 0.003, indicating minimal variation. This consistency suggests that common method bias does not pose a significant threat to the validity of our findings, and the model can be considered robust. See **Table 1**.

**Table 1:** Comparison of path coefficients before and after controlling for the marker variable.

Path	Original model *β*		Model controlling for marker *β*		Change (Δ|*β*|)
	Effect (*β*)	Bootstrap 95% CI	Effect (*β*)	Bootstrap 95% CI	
A: Neglectful Parenting Model					
Impulsivity→NSSI(Direct effect)	0.154	-0.002˜0.31	0.153	-0.004˜0.309	0.001
Impulsivity→Negative Parenting Styles→NSSI	0.026	-0.017˜0.082	0.025	-0.024˜0.073	0.001
Impulsivity→Distress Tolerance→NSSI	0.038	0.004˜0.113	0.038	0.000˜0.099	0.000
Impulsivity→Negative Parenting Styles→Distress Tolerance→NSSI	0.023	0.007˜0.052	0.023	0.004˜0.046	0.000
Total indirect effect	0.087	0.036˜0.172	0.086	0.027˜0.154	0.001
Total effect	0.241	0.086˜0.396	0.238	0.083˜0.394	0.003
B: Positive Parenting Model					
Impulsivity→NSSI(Direct effect)	0.138	-0.018˜0.295	0.138	-0.018˜0.295	0.000
Impulsivity→Positive Parenting Styles→ NSSI	0.035	0.006˜0.092	0.035	0.000˜0.078	0.001
Impulsivity→Distress Tolerance→NSSI	0.058	0.017˜0.132	0.058	0.012˜0.121	0.000
Impulsivity→Positive Parenting Styles→ Distress Tolerance→NSSI	0.008	0.001˜0.026	0.008	0.000˜0.021	0.000
Total indirect effect	0.102	0.046˜0.201	0.100	0.036˜0.180	0.002
Total effect	0.241	0.086˜0.396	0.238	0.083˜0.394	0.003

## 3 Results

### 3.1 Descriptive statistics with correlation analyses

The demographic data of the cases is presented in **Table 2**. The analysis results indicate that, after controlling for sociodemographic parameters, the partial correlations among the variables are presented in **Table 3**.

**Table 2:** Descriptive statistics (N = 311).

Variable	Category	N	(%)
Gender	Male	112	36
	Female	199	64
Grade	Seventh grade	121	38.9
	Eighth grade	70	22.5
	Ninth grade	34	10.9
	Tenth grade	54	17.4
	Eleventh grade	25	8
	Twelfth grade	7	2.3
Only- children	Yes	102	32.8
	No	209	67.2
Divorced family	Yes	55	17.7
	No	256	82.3
Father’s education	College or higher	142	45.7
	Senior secondary	87	28
	Junior high school	59	19
	Primary school or lower	23	7.4
Mother’s education	College or higher	137	44.1
	Senior secondary	77	24.8
	Junior high school	63	20.3
	Primary school or lower	34	10.9

**Table 3:** Descriptive analysis and correlations (N = 311).

Variable	(*M* ± *SD*)	NSSI	Impulsivity	Distress tolerance	Negative parenting styles	Positive parenting styles
NSSI	15.96 ± 18.62	1				
Impulsivity	75.67 ± 13.61	0.17**	1			
Distress Tolerance	35.35 ± 10.24	-0.26***	-0.2***	1		
Negative Parenting Styles	4.08 ± 1.01	0.18**	0.19***	-0.39***	1	
Positive Parenting Styles	4.99 ± 1.50	-0.16**	-0.22***	0.16**	-0.17**	1
Father’s Educational Attainment (Coded Variable)	1.88 ± 0.965	0.036	0.095	-0.037	0.040	-0.217***

** indicates *p* < 0.01; *** indicates *p* < 0.001.

The results of the independent sample t-test revealed a significant difference in positive parental rearing based on gender (*t* = 1.991, *p* < 0.05) and a notable difference in negative extreme parental rearing related to parental divorce status (*t* = -4.324, *p* < 0.001). Additionally, there was a significant difference in positive parenting concerning being an only child *(t* = 4.304, *p* < 0.001). One-way ANOVA results indicated that the BIS-11 scores varied significantly by grade (*F* = 2.29, *p* < 0.05). Subsequent multiple *post-hoc* comparisons and LSD tests demonstrated that the BIS-11 scores of senior three students were significantly higher than those of junior seven and junior two students.

### 3.2 The chain mediating effects analyses

**Figure 3:**
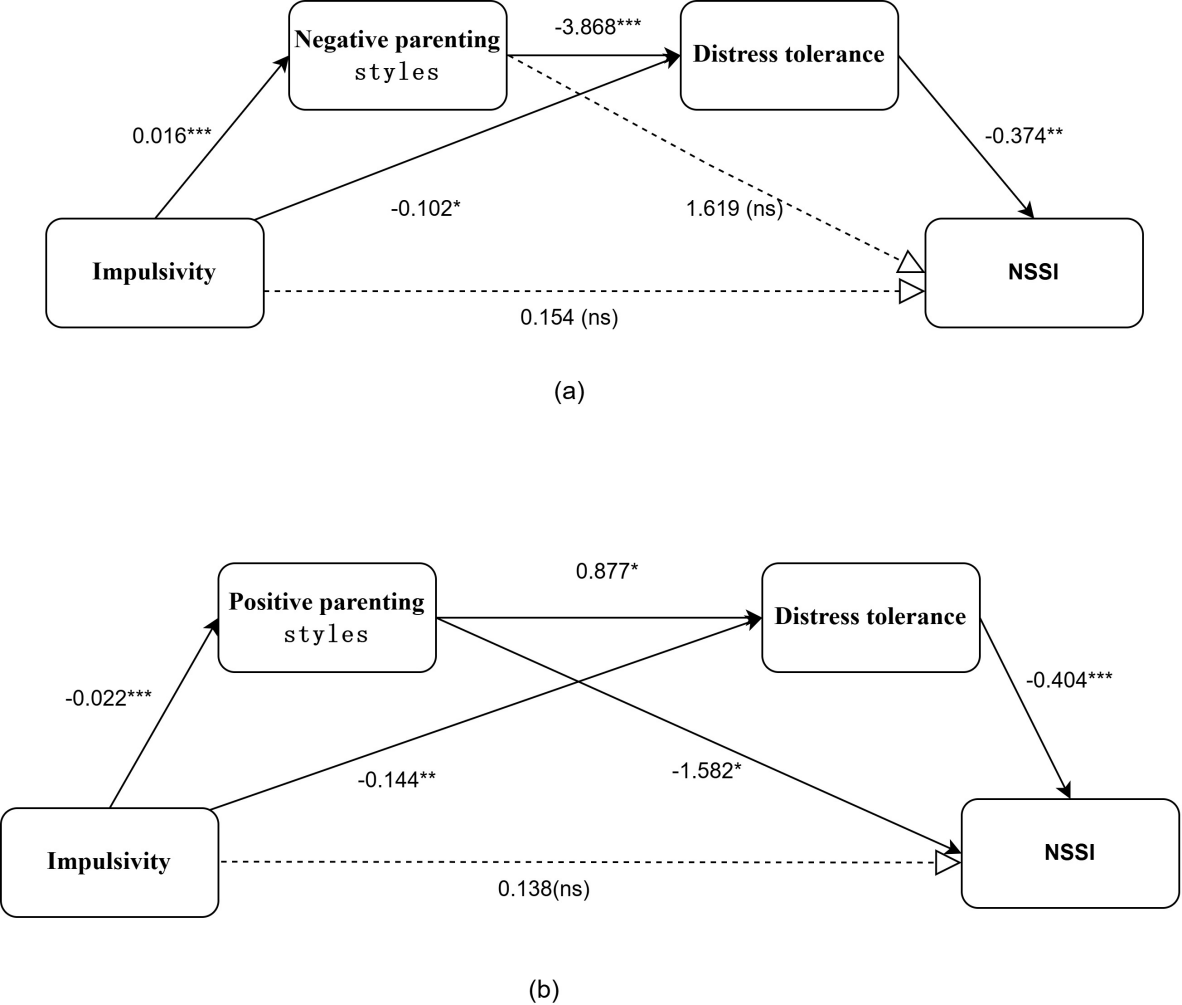
Path coefficient diagram of the chain agency model. **(a)** Negative parenting model. **(b)** Positive parenting model. *p < 0.05; **p < 0.01; ***p < 0.001;Dotted lines indicate paths that are not statistically significant (ns); NSSI denotes non-suicidal self-injury.

The chained mediation model examining the relationship between impulsivity and NSSI was assessed using Model 6 of the PROCESS macro in SPSS to evaluate chained multiple mediation models. After controlling for gender, grade, only-child status, and parental divorce, we established a model with impulsivity as the independent variable, NSSI as the dependent variable, and parenting styles (both negative and positive) along with distress tolerance as mediating variables. We respective evaluated the chained mediation effects in negative and positive parenting styles models. Utilizing the Bootstrap method, we drew 5,000 repeated samples to assess the mediating effects, maintaining a 95%CI. The diagrams illustrating the chained mediating effects are presented in **Figure 3**.

As demonstrated in Model 1 (**Table 4**), When negative parenting styles and distress tolerance serve as mediating variables, the chain mediating effect comprises two distinct pathways. The first pathway, “impulsivity → distress tolerance → NSSI,” yields a mediating effect value of 0.038, representing 15.77% of the total effect size. The second pathway, “impulsivity → negative parenting styles→ distress tolerance → NSSI,” produces a mediating effect value of 0.023, accounting for 9.54% of the total effect size.

**Table 4:** Model effect decomposition and bootstrap test results.

Model and effect type	Path	Effect (*β*)	SE	Bootstrap 95% CI
A: Neglectful Parenting Model				
Simple Mediation Model	Impulsivity→Negative Parenting Styles→NSSI	0.026	0.024	-0.017˜0.082
Simple Mediation Model	Impulsivity→Distress Tolerance→NSSI	0.038	0.026	0.004˜0.113
Chain-based Intermediary Model	Impulsivity→Negative Parenting Styles→Distress Tolerance→NSSI	0.023	0.011	0.007˜0.052
Direct effect	Impulsivity→NSSI	0.154	0.079	-0.002˜0.31
Total indirect effect	Sum of all indirect paths	0.087	0.033	0.036˜0.172
Total effect	Impulsivity→NSSI (uncontrolled intermediaries)	0.241	0.079	0.086˜0.396
B: Positive Parenting Model				
Simple Mediation Model	Impulsivity→Positive Parenting Styles→ NSSI	0.036	0.021	0.006˜0.092
Simple Mediation Model	Impulsivity→Distress Tolerance→NSSI	0.058	0.029	0.017˜0.132
Chain-based Intermediary Model	Impulsivity→Positive Parenting Styles→ Distress Tolerance→NSSI	0.008	0.006	0.001˜0.026
Direct effect	Impulsivity→NSSI	0.138	0.079	-0.018˜0.295
Total indirect effect	Sum of all indirect paths	0.102	0.037	0.046˜0.201
Total effect	Impulsivity→NSSI (uncontrolled intermediaries)	0.241	0.079	0.086˜0.396

Model 2 (see **Table 4**) revealed that positive parenting styles and distress tolerance serve as mediating variables, the chain mediating effect comprises three distinct pathways. The mediating effect arising from the pathway “impulsivity → positive parenting styles→ NSSI” has an effect value of 0.036, which represents 14.93% of the total effect size. The mediating effect from the pathway “impulsivity → distress tolerance → NSSI” has an effect value of 0.058, accounting for 24.07% of the total effect size. Lastly, the mediating effect generated by the pathway “impulsivity → positive parenting styles → distress tolerance → NSSI” has an effect value of 0.008, constituting 3.32% of the total effect size.

## 4 Discussion

### 4.1 The direct effect of impulsivity on NSSI was not significant

This study investigates the psychosocial pathways through which impulsivity predicts NSSI. As anticipated, the chain mediating effect is significant, thereby supporting the theoretical model. However, an additional noteworthy finding is that the direct effect is not significant, which contradicts previous research (27). This discrepancy may be attributed to differences in model specification and analytical approach. While Cassels et al. examined impulsivity as an independent predictor of new-onset NSSI in a prospective longitudinal design without testing mediating pathways, the present study incorporated parenting styles and distress tolerance as sequential mediators in a cross-sectional mediation model. The significant direct effect of impulsivity observed in their study may reflect the absence of these intervening variables in their analytical framework; once parenting and distress tolerance are accounted for, the impulsivity-NSSI association appears to be fully transmitted through these psychosocial pathways. Additionally, Cassels et al. found that psychological distress mediated the parenting-NSSI association, which aligns with our finding that distress tolerance serves as a key mechanism, further supporting the theoretical plausibility of the chain mediation model tested here.

Drawing on social neuroscience, Steinberg proposed that the heightened prevalence of risk-taking behaviors in adolescence arises from a ‘developmental lag,’ in which the socioemotional system matures earlier than the cognitive control system. During early adolescence, the limbic system exhibits heightened activity due to hormonal influences, while the maturation of the prefrontal cortex extends into later youth. This “developmental jet lag” results in adolescents typically exhibiting neural characteristics associated with high emotional arousal and diminished impulse control (28). However, this neurodevelopmental context does not inherently lead to NSSI behavior; it must be activated through specific functional mechanisms. According to the functional model of NSSI (29), NSSI is primarily maintained by its automatic negative reinforcement function—the immediate relief from aversive emotional states. Adolescents with heightened emotional arousal and diminished impulse control are particularly sensitive to such immediate affective changes. When the family environment fails to provide adequate emotion regulation support, these individuals lack alternative strategies to manage distress. In this context, impulsivity increases the likelihood of NSSI not directly, but through the mediating pathway of emotion regulation difficulties, by heightening reliance on NSSI’s negatively reinforcing effects (30). Thus, the interaction between neurodevelopmental vulnerability and environmental deficits manifests in the adoption of NSSI as a maladaptive but functionally reinforced emotion regulation strategy.

According to the findings of this study, negative parenting styles co-occur with an increased risk of maladaptive behavior in individuals with this vulnerable neural foundation. In contexts where the family environment fails to provide adequate emotional regulation support, high impulsivity is more likely to be associated with actual NSSI through this mediating pathway. Consequently, this finding necessitates a re-evaluation of the role of impulsivity in the development of NSSI. Rather than serving as a proximal trigger for NSSI behavior, impulsivity appears to function as a distal susceptibility factor. The results revealed a full mediation pattern: impulsivity did not have a significant direct effect on NSSI after accounting for the mediators. Instead, its statistical association with NSSI was entirely accounted for by the sequential pathway of parenting styles and distress tolerance. This finding suggests that the link between impulsivity and NSSI can be understood in terms of how impulsive individuals experience parenting and subsequently develop distress tolerance, rather than operating through other unmeasured mechanisms involving impulsivity itself. This study aims not only to test the mediating pathway but also to provide empirical evidence that the total effect of impulsivity on NSSI is entirely transmitted through a chain mediating model. This approach more clearly delineates the specific pathway through which impulsivity is associated with NSSI. This approach more clearly delineates the role of impulsivity in the development of NSSI. Thus, the full mediation observed in this study is not a statistical artifact but a theoretically grounded finding that underscores the distal and conditional nature of impulsivity as a risk factor for NSSI.

### 4.2 The mediating role of distress tolerance

The observed results align with the DSM-5 diagnostic criteria for NSSI, which suggest that interpersonal difficulties or negative emotions frequently precede NSSI. This study demonstrates that impulsivity is associated with adolescent NSSI through the mediating role of distress tolerance. When adolescents encounter intense negative emotions, those with low distress tolerance exhibit heightened emotional susceptibility, inadequate emotional regulation, and challenges in enduring painful feelings. In such instances, highly impulsive teenagers are inclined to pursue immediate solutions to alleviate their negative emotions, with self-harm serving as one such strategy. This finding aligns with the experience avoidance model proposed by Chapman et al. (31), which posits that individuals quickly seek to evade negative emotional experiences triggered by both internal and external stimuli. In comparison to adolescents without a history of NSSI, those with such a history exhibit reduced emotional sensitivity and impaired inhibitory control when confronting negative emotions (4; J. 32). This finding aligns with the theoretical framework suggesting that NSSI behavior can promote endorphin release, resulting in temporary relief (33).

### 4.3 The mediating role of parenting styles

This study identified a positive correlation between NSSI behavior and negative parenting styles characterized by overprotection and exclusion, while revealing a negative correlation with positive parenting styles that emphasize emotional warmth. Additionally, impulsivity exhibited a negative correlation with positive parenting styles and a positive correlation with negative parenting styles. These findings indicate that impulsivity is associated with individuals’ perceptions of both positive and negative parenting styles. Further mediation analysis revealed that positive parenting styles mediated the association between impulsivity and NSSI. Specifically, emotional warmth within parenting practices was linked to lower NSSI engagement among adolescents, supporting the protective role of positive parenting. Emotional warmth refers to parents’ sensitivity to their children’s emotional fluctuations, as well as the care and protection they provide. When children face difficulties or setbacks, it is essential for parents to offer support and encouragement. Prior research on adolescents has demonstrated that parental emotional warmth is negatively associated with NSSI behavior and serves as a protective factor for these individuals (14, 34).

In contrast, although negative parenting styles showed significant bivariate correlations with both impulsivity and NSSI, they did not emerge as significant mediators in the chain model. This finding aligns with the “protective-protective model” (35), which suggests that protective factors (e.g., positive parenting) can buffer the effects of risk factors, whereas risk factors alone do not necessarily activate a risk pathway in the absence of other vulnerabilities. Thus, the pathway from impulsivity to NSSI may operate primarily through the presence of protective parenting factors rather than the absence of risk factors.

### 4.4 Overall chain-based intermediary model

This study presents a dual-path integration model for the impulsive prediction of NSSI. According to Bronfenbrenner’s ecosystem theory, the family serves as the micro-environment with which individuals directly engage, representing the initial and most fundamental context influencing development (36). The overall atmosphere of the family environment and the quality of parent-child interactions profoundly affect the formation and development of an individual’s personality (37). Furthermore, the parenting style adopted by parents is considered a crucial variable that determines the emotional tone of parent-child interactions, thereby fundamentally shaping the social and emotional backdrop of the family (38). Consequently, it is posited that family parenting styles, as early environmental factors, precede the development of distress tolerance. In the negative parenting model, the pathway from impulsivity to negative parenting style to NSSI was not significant. This indicates that the mediating role of negative parenting in the impulsivity-NSSI association was contingent upon the inclusion of distress tolerance in the sequential model. The association between negative parenting and NSSI was only observed when negative parenting styles further are associated with lower distress tolerance.

In the positive parenting model, all pathways were significant, with the exception of the direct effect of impulsivity on NSSI, which was not significant. This finding supports a full mediation model, wherein the relationship between impulsivity and NSSI was accounted for by the sequential effects of positive parenting and distress tolerance. The pattern of associations suggests that positive parenting is linked to higher distress tolerance, which in turn is related to lower NSSI, highlighting the potential protective role of positive parenting in this pathway.

While these findings are consistent with the proposed theoretical model, causal conclusions cannot be drawn due to the cross-sectional design. If future research confirms these relationships, interventions aimed at enhancing positive parenting and distress tolerance may help reduce NSSI risk among highly impulsive adolescents.

High impulsivity, when coupled with low distress tolerance, serves as a significant risk factor for NSSI in adolescents. Both impulsivity and distress tolerance are critical cross-diagnostic factors (39, 40). Furthermore, high impulsivity is a notable characteristic among adolescents with gaming disorder (41), which may help explain the frequent comorbidity of NSSI with other mental health disorders, including depression, anxiety, and gaming disorder. The role of impulsivity can be initially characterized by its association with lower family relationship quality and lower distress tolerance, ultimately increasing the likelihood of NSSI. Consequently, impulsivity emerges as a distal vulnerability factor that may be shaped by environmental factors through psychological resources. This conclusion addresses the inconsistencies observed in previous studies concerning the direct effects of impulsivity.

This model was supported in a sample of adolescents without comorbid mental disorders, thereby facilitating the identification of a more distinct mediating pathway in the relationship between impulsivity and NSSI. It is important to note that these findings are based on cross-sectional data and reflect statistical mediation rather than causal processes. While the observed patterns are consistent with the proposed theoretical model, causal inferences cannot be drawn from this study. The terms ‘pathway’ and ‘mediator’ are used in a statistical sense to describe associations among variables, and do not imply temporal or causal relationships

### 4.5 Limitations and future directions

This study employs a cross-sectional design, which constrains our ability to draw causal inferences from the data. Chain mediation analysis does not establish causal relationships. Future research utilizing longitudinal or intervention designs is essential for confirming the causal directions among these pathways. Additionally, the insignificance of the direct path coefficient observed in this study may be affected by sample characteristics and measurement methods. Future investigations should assess the stability of this fully mediating model across diverse populations and compare clinical and non-clinical samples to determine whether the impulsive action mode varies with the severity of the pathology. Furthermore, although this study implemented a multi-faceted approach—including programmed control, Harman’s single-factor test, and the labeled variable method (father’s educational level)—to mitigate the influence of common method bias, the inherent limitations of cross-sectional self-reported data remain. Future research could address this issue by incorporating informant reporting and longitudinal designs. This sample is concentrated in Guiyang, China, and is restricted to a single region, with 64% of the population being female. Such an unbalanced gender distribution may influence the interpretation of the results. Additionally, the samples did not encompass rural families across various economic levels, which restricts the generalizability of the conclusions. Furthermore, the measurement dimensions of negative parenting styles in this study are relatively narrow, primarily focusing on “overprotection” and “rejection,” while neglecting other critical negative parenting behaviors such as “emotional neglect” and “severe punishment.” This limitation may result in an incomplete assessment of negative parenting constructs, potentially underestimating the extent of their indirect effects within the chain model. Future research should incorporate multi-dimensional parenting style questionnaires to more thoroughly investigate the mediating role of family factors in the ‘impulsivity → NSSI’ pathway and examine whether different parenting dimensions exert distinct influence mechanisms.

Third, this study focused on mediating mechanisms and did not examine potential moderating variables that might influence the relationships among impulsivity, parenting styles, distress tolerance, and NSSI. For example, academic stress and peer relationships—two factors closely linked to adolescent development—may moderate these pathways. Academic stress could exacerbate the impact of negative parenting on distress tolerance, while positive peer relationships might buffer the effects of impulsivity on NSSI. Future research should incorporate such moderators to test whether the proposed mediation model operates differently across varying levels of these contextual factors. Identifying moderating variables would also help refine intervention strategies by specifying for whom and under what conditions the observed pathways are most salient.

## 5 Conclusion

This study found that, among adolescents engaging in NSSI without other mental disorders, the relationship between impulsivity and NSSI was fully mediated by parenting styles and distress tolerance. Higher impulsivity was associated with more negative parenting and less positive parenting; negative parenting was associated with lower distress tolerance, while positive parenting was associated with higher distress tolerance; and lower distress tolerance was in turn associated with higher NSSI. This pattern indicates that impulsivity is not a direct or strong predictor of NSSI, but rather a distal factor whose influence on NSSI is conditional upon parenting styles and distress tolerance. Distress tolerance emerged as the most proximal correlate of NSSI, suggesting it may be a key mechanism through which both individual and familial factors are linked to NSSI behavior.

From a statistical perspective, negative parenting and lower distress tolerance emerged as risk correlates (associated with higher NSSI), while positive parenting and higher distress tolerance emerged as protective correlates (associated with lower NSSI). If future longitudinal research confirms these relationships as causal, interventions could target distress tolerance as a key mechanism and promote positive parenting practices to reduce NSSI risk.

## Data Availability

The datasets presented in this study can be found in online repositories. The names of the repository/repositories and accession number(s) can be found below: https://figshare.com/articles/dataset/Data_on_non-suicidal_self-injury_among_adolescents/30879659?file=60346199.
